# Serum inflammatory cytokines predict 90-day outcome after endovascular treatment in acute ischemic stroke

**DOI:** 10.3389/fneur.2026.1903651

**Published:** 2026-08-26

**Authors:** Guo-Qing Zhao, Zi-Ai Zhao, Xiao-Tong Tang, Wei Zhang, Hui-Sheng Chen

**Affiliations:** 1 Department of Neurology, General Hospital of Northern Theater Command, Shenyang, China; 2 Department of Clinical Laboratory Medicine, General Hospital of Northern Theater Command, Shenyang, China; 3 Department of Internal Medicine, The 965th Hospital of Chinese PLA, Jilin, China

**Keywords:** acute ischemic stroke, endovascular treatment, inflammatory cytokines, interleukin-10, interleukin-6, prognostic model

## Abstract

**Background and objective:**

Inflammation plays a critical role in acute ischemic stroke (AIS) pathophysiology and may influence outcomes after endovascular treatment (EVT). However, the incremental prognostic value of inflammatory cytokines beyond conventional predictors remains unclear. This study aimed to investigate the association of cytokines with 90-day functional outcomes in EVT-treated AIS patients and evaluate their value in prognostic modeling.

**Methods:**

Based on the prospective DETECT2-China registry, we included consecutive large vessel occlusion patients undergoing EVT between March 2022 and March 2025. Plasma cytokines were measured within 24 h post-EVT. The primary outcome was 90-day poor functional outcome (modified Rankin Scale [mRS] 3–6). Multivariable logistic regression identified independent predictors. Clinical, cytokine, and combined models were constructed and evaluated using receiver operating characteristic (ROC) curves, calibration, decision curve analysis (DCA), and reclassification metrics.

**Results:**

Among 139 included patients, 77 (55.4%) had 90-day poor outcomes. Serum IL-6 and IL-10 levels were significantly higher in these patients. In multivariable analysis, log₂-IL-6 was an independent predictor (OR = 1.43 per doubling, 95% CI: 1.14–1.79, *p* = 0.002), and log_2_-IL-10 was significant in Adjusted Model 2 (OR = 1.86 per doubling, 95% CI: 1.02–3.36, *p* = 0.041). The combined model achieved superior discrimination (AUC: 0.841, 95% CI: 0.770–0.905) compared with the clinical model (AUC: 0.791, 95% CI: 0.717–0.865; *p* = 0.015), with good calibration and clinical net benefit. Reclassification was also improved (continuous NRI: 43.32%, *p* = 0.009; IDI: 7.27%, *p* < 0.001).

**Conclusion:**

Serum IL-6 is independently associated with 90-day poor outcome While incorporating cytokines into clinical models may improve prognostic estimation, these exploratory findings require validation in independent cohorts before clinical application.

## Introduction

Acute ischemic stroke (AIS) remains one of the leading causes of mortality and long-term disability worldwide. Although advances in reperfusion therapies, particularly endovascular treatment (EVT), have significantly improved recanalization rates and clinical outcomes, a substantial proportion of patients still fail to achieve favorable functional recovery despite successful vessel reopening (1, 2). This discrepancy suggests that post-stroke outcomes are influenced not only by recanalization status but also by complex secondary pathophysiological processes that are not fully captured by conventional clinical indicators.

Inflammation has been increasingly recognized as a central mechanism in the pathophysiology of AIS and a critical determinant of clinical outcomes. Following cerebral ischemia, a rapid cascade of neuroinflammatory responses—involving the release of a broad spectrum of cytokines and inflammatory mediators—contributes to blood–brain barrier disruption and secondary neuronal injury, which may be further amplified by reperfusion after EVT (3–5). Consequently, peripheral inflammatory cytokines have emerged as promising biomarkers. These circulating mediators reflect the magnitude of ischemic injury and systemic immune responses, closely associating with infarct progression and long-term functional outcomes (6, 7). However, current studies have reported heterogeneous results regarding the prognostic value of individual cytokines, likely due to differences in study design, patient populations, and timing of biomarker measurement. Moreover, most investigations have focused on single biomarkers rather than evaluating their combined effects within a comprehensive predictive framework.

Although several prognostic models incorporating clinical variables or peripheral blood biomarkers have been proposed, their predictive performance remains suboptimal. Most existing models rely primarily on baseline clinical characteristics or imaging findings, with limited integration of upstream inflammatory mediators that reflect dynamic biological processes following reperfusion (8).

Therefore, the present study aimed to investigate the association between inflammatory cytokines and 90-day functional outcomes in AIS patients undergoing EVT and to develop a predictive model integrating clinical variables and inflammatory biomarkers. Furthermore, we systematically evaluated the incremental predictive value of inflammatory markers using discrimination, calibration, and reclassification metrics to support biomarker-guided risk stratification.

## Methods

### Study design and participants

This study was derived from the DETECT2-China registry, a prospective multicenter real-world study designed to evaluate the effectiveness and safety of EVT in patients with AIS in China. Specifically, the current study represents a single-center analysis of patient data from a single participating center (the General Hospital of Northern Theater Command) where systematic baseline serum inflammatory cytokine measurements were performed. The study protocol was approved by the Institutional Review Board of the General Hospital of Northern Theater Command and registered at ClinicalTrials.gov (NCT05092139).

From this registry, we screened consecutive patients with LVO who underwent EVT at our stroke center between March 2022 and March 2025. The inclusion criteria were as follows: (1) age ≥ 18 years; (2) AIS confirmed by computed tomography (CT) or magnetic resonance imaging (MRI); (3) patients receiving EVT; (4) pre-stroke modified Rankin Scale (mRS) ≤ 2; (5) availability of inflammatory cytokine measurements; and (6) provision of written informed consent. The exclusion criteria were: (1) presence of intracranial hemorrhage on baseline imaging; (2) pre-stroke mRS ≥ 3; (3) severe comorbidities such as active malignancy, end-stage renal disease, or severe liver dysfunction that could confound 90-day outcomes; and (4) incomplete clinical or inflammatory cytokine measurements.

### Data collection and definitions

Baseline demographic characteristics, vascular risk factors, and clinical data were systematically collected, including age, sex, hypertension, diabetes mellitus, smoking and drinking status, and baseline neurological severity assessed by the National Institutes of Health Stroke Scale (NIHSS) (9). Blood pressure, heart rate, and workflow time metrics were also recorded. Recanalization status was evaluated using the modified Thrombolysis in Cerebral Infarction (mTICI) scale, with successful recanalization defined as mTICI 2b–3. Functional outcome was assessed using the modified Rankin Scale (mRS) at 90 days (10), obtained through standardized telephone interviews or outpatient follow-up by certified neurologists. Good functional outcome was defined as mRS 0–2, and poor functional outcome as mRS 3–6 (11).

### Measurement of inflammatory cytokines

Plasma samples were collected within 24 h following endovascular treatment (EVT). The concentrations of inflammatory cytokines were quantified using multiplex microsphere flow immunofluorescence. The cytokine detection kits were provided by Raisecare Biotechnology Co., Ltd. (Shandong, China). Briefly, 25 μL of experimental buffer, 25 μL of centrifuged plasma, 25 μL of capture microsphere antibodies, and 25 μL of detection antibodies were precisely dispensed into flow cytometry tubes. The mixtures were incubated at room temperature in the dark for 2 h with gentle agitation. Subsequently, 25 μL of streptavidin-phycoerythrin (SA-PE) was added, and the incubation was continued for an additional 30 min. Each tube was then supplemented with 500–1,000 μL of 1 × wash buffer, vortexed briefly, and centrifuged at 300–500 × g for 5 min. The supernatant was carefully decanted, and the tubes were inverted onto absorbent paper to remove residual liquid. The microsphere pellets were then resuspended in 150–300 μL of wash buffer by vortexing for 10 s. Immediately following resuspension, the samples were loaded onto a flow cytometer for detection.

### Statistical analysis

Continuous variables were expressed as median (interquartile range, IQR), depending on data distribution. Group comparisons were performed using Student’s *t*-test or Mann–Whitney *U* test as appropriate. Categorical variables were expressed as counts (percentages) and compared using the chi-square (*χ*^2^) test or Fisher’s exact test.

**Figure 1:**
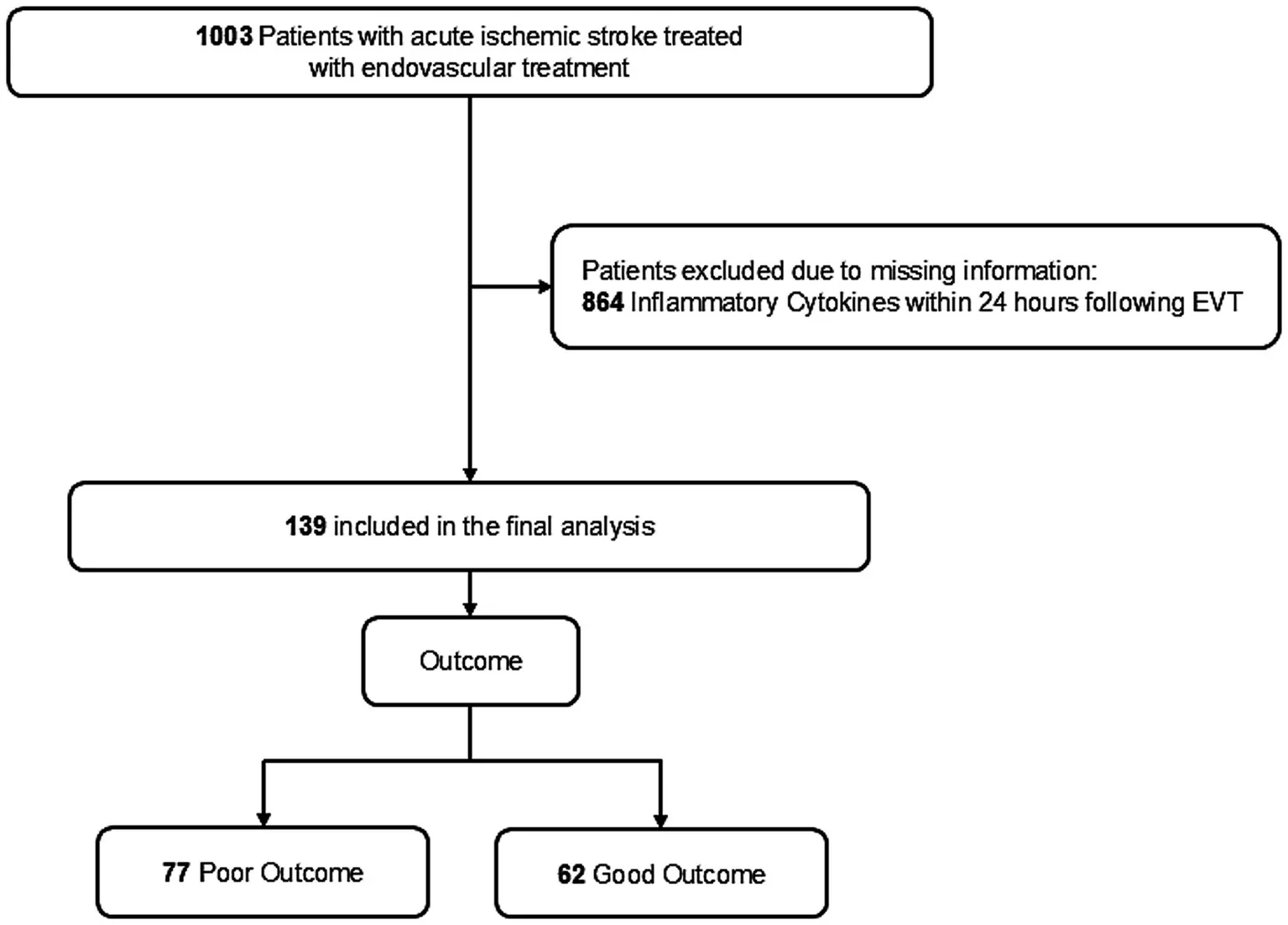
Flowchart of patient selection. A total of 1,003 patients with acute ischemic stroke treated with endovascular treatment were initially screened. After excluding 864 patients with missing data on inflammatory cytokines within 24 h following EVT, 139 eligible patients were included in the final analysis. Based on the 90-day modified Rankin Scale (mRS), the cohort was categorized into a poor functional outcome group (*n* = 77) and a good functional outcome group (*n* = 62).

Patients with missing cytokine measurements were excluded, and the analysis was performed using complete-case analysis (see Figure 1 for the number of excluded patients).

Univariate and multivariable logistic regression analyses were conducted to identify independent predictors of 90-day poor functional outcome. Variables with clinical relevance or *p* < 0.05 in univariate analysis were included in multivariable models. For continuous biomarkers (IL-6 and IL-10), the variables were log₂-transformed prior to regression analyses to account for their right-skewed distributions, and the odds ratios were calculated per 1-unit increase (representing a doubling of the cytokine concentration) to facilitate clinical interpretation. Results were reported as odds ratios (ORs) with 95% confidence intervals (CIs). To minimize the risk of overfitting, the number of candidate predictors included in the multivariable models was restricted according to the events-per-variable (EPV) principle. In our main adjusted models, a maximum of 7 predictors were included. Based on the smaller outcome group (*n* = 62, good functional outcome), the EPV was approximately (8, 9), which is close to the generally recommended threshold of 10. Furthermore, internal validation using 1,000 bootstrap resamples was performed not only to evaluate model calibration but also to calculate the optimism-corrected area under the curve (AUC) as a sensitivity analysis, thereby providing a more realistic estimate of the model’s predictive performance. Multicollinearity among covariates in the multivariable models was evaluated using the variance inflation factor (VIF).

Three models were constructed: a clinical model, a cytokine model, and a combined model integrating both. Model performance was assessed using receiver operating characteristic (ROC) curves and area under the curve (AUC), with comparisons performed using the DeLong test. Because only two models (clinical vs. combined) were formally compared, no adjustment for multiple comparisons was applied. Calibration was evaluated using the Hosmer–Lemeshow goodness-of-fit test and calibration curves. Clinical utility was assessed by decision curve analysis (DCA). Net reclassification improvement (NRI) and integrated discrimination improvement (IDI) were calculated to quantify the improvement in risk prediction after adding inflammatory cytokines to the clinical model.

All statistical analyses were performed using SPSS (version 26.0, IBM Corp.) and R software (version 4.1.0). Two-sided *p* values < 0.05 were considered statistically significant.

## Results

### Patient characteristics

A total of 139 patients with acute ischemic stroke who underwent endovascular treatment (EVT) were included in the final analysis (Figure 1). At 90 days, 62 patients (44.6%) achieved a good functional outcome (modified Rankin Scale [mRS] 0–2), while 77 patients (55.4%) had a poor functional outcome (mRS 3–6).

Baseline clinical characteristics and inflammatory cytokine levels are summarized in Table 1. Compared with patients with good functional outcome, those with poor functional outcome were significantly older (median 69.0 vs. 61.5 years, *p* < 0.001) and had a higher prevalence of diabetes (32.5% vs. 11.3%, *p* = 0.004). They also presented with higher baseline NIHSS scores (16.0 vs. 14.0, *p* = 0.002) and elevated systolic blood pressure on admission (146.0 vs. 133.5 mmHg, *p* = 0.011). In contrast, the rate of successful recanalization was significantly lower in the poor functional outcome group (83.1% vs. 96.8%, *p* = 0.012).

**Table 1:** Baseline characteristics of patients.

Variable	Overall (*N* = 139)	Good functional outcome (*N* = 62)	Poor functional outcome (*N* = 77)	*p* value
Age, median (IQR), years	66.00 (58.00, 72.00)	61.50 (53.00, 67.00)	69.00 (61.00, 76.00)	<0.001
Male, No. (%)	101 (72.66%)	49 (79.03%)	52 (67.53%)	0.180
Risk factors, No. (%)				
Current smoker	71 (51.08%)	37 (59.68%)	34 (44.16%)	0.088
Current drinker	30 (21.58%)	20 (32.26%)	10 (12.99%)	0.007
Prior stroke or TIA	40 (28.78%)	14 (22.58%)	26 (33.77%)	0.188
Hypertension	88 (63.31%)	36 (58.06%)	52 (67.53%)	0.290
Diabetes	32 (23.02%)	7 (11.29%)	25 (32.47%)	0.004
Ischemic heart disease	4 (2.88%)	2 (3.23%)	2 (2.60%)	0.999
Atrial fibrillation	32 (23.02%)	10 (16.13%)	22 (28.57%)	0.106
Baseline NIHSS, median (IQR)	15.00 (12.00, 19.00)	14.00 (11.00, 18.00)	16.00 (13.00, 21.00)	0.002
Baseline SBP, median (IQR)	139.00 (124.00, 161.00)	133.50 (121.00, 153.00)	146.00 (129.00, 165.00)	0.011
Baseline DBP, median (IQR)	82.00 (75.00, 95.00)	79.00 (73.00, 91.00)	84.00 (75.00, 95.00)	0.209
Baseline HR, median (IQR)	72.00 (62.00, 80.00)	70.00 (60.00, 77.00)	72.00 (63.00, 84.00)	0.161
Intravenous thrombolysis, No. (%)	31 (22.30%)	16 (25.81%)	15 (19.48%)	0.416
Workflow times, median (IQR), min				
OPT	492.00 (347.00, 794.00)	493.50 (337.00, 750.00)	492.00 (352.00, 837.00)	0.869
ORT	532.00 (400.00, 760.00)	532.00 (390.00, 760.00)	532.00 (440.00, 755.00)	0.668
PRT	55.00 (40.00, 70.00)	51.50 (35.00, 70.00)	55.00 (46.00, 69.00)	0.087
Successful recanalization, *n* (%)	124 (89.21%)	60 (96.77%)	64 (83.12%)	0.012
Stroke etiology, No. (%)^§^				
LAA	101 (72.66%)	48 (77.42%)	53 (68.83%)	0.383^§^
CE	37 (26.62%)	14 (22.58%)	23 (29.87%)	
Other	1 (0.72%)	0 (0.00%)	1 (1.30%)	
Cytokines, median (IQR)				
IL-1β	3.42 (1.19, 8.52)	3.25 (1.19, 8.52)	3.61 (1.30, 8.52)	0.263
IL-2	1.64 (1.35, 2.23)	1.61 (1.30, 2.14)	1.65 (1.38, 2.26)	0.228
IL-4	0.97 (0.91, 1.76)	0.97 (0.91, 1.76)	0.98 (0.91, 1.76)	0.812
IL-5	1.84 (1.82, 1.94)	1.82 (1.82, 1.98)	1.84 (1.82, 1.93)	0.777
IL-6	11.02 (4.51, 35.37)	6.56 (2.99, 13.36)	17.53 (7.41, 61.57)	<0.001
IL-8	8.66 (2.23, 23.58)	7.41 (2.23, 13.28)	11.53 (2.23, 27.20)	0.092
IL-10	2.23 (1.75, 3.10)	2.05 (1.60, 2.41)	2.41 (1.89, 5.80)	<0.001
IL-12p70	1.47 (1.19, 1.90)	1.47 (1.18, 2.12)	1.45 (1.21, 1.81)	0.959
IL-17	2.17 (2.12, 3.06)	2.14 (2.11, 2.71)	2.27 (2.14, 3.59)	0.188
TNF-α	1.49 (1.49, 2.05)	1.49 (1.49, 1.87)	1.49 (1.49, 2.05)	0.369
IFN-α	1.66 (1.61, 2.12)	1.75 (1.61, 2.29)	1.61 (1.61, 2.01)	0.137
IFN-γ	2.08 (2.05, 4.15)	2.17 (2.05, 4.54)	2.05 (2.05, 3.90)	0.476

Data were expressed as median (IQR) or *N* (%). CE, cardioembolism; DBP, diastolic blood pressure; HR, heart rate; IFN, interferon; IL, interleukin; LAA, large artery atherosclerosis; NIHSS, National Institutes of Health Stroke Scale; OPT, onset-to-puncture time; ORT, onset-to-recanalization time; PRT, puncture-to-recanalization time; SBP, systolic blood pressure; TIA, transient ischemic attack; TNF, tumor necrosis factor.

^§^Calculated using Fisher’s exact test due to small cell size.

Among inflammatory cytokines, serum IL-6 and IL-10 levels were markedly higher in patients with poor functional outcome (IL-6: 17.53 vs. 6.56, *p* < 0.001; IL-10: 2.41 vs. 2.05, *p* < 0.001), whereas other cytokines showed no statistically significant differences.

### Independent predictors of 90-day poor functional outcome

Univariate and multivariable logistic regression analyses were performed to identify independent predictors of poor functional outcome (Table 2). Variables with clinical relevance and statistical significance were included in multivariable models, with careful consideration of the events-per-variable principle to minimize overfitting.

**Table 2:** Binary logistic regression analysis of factors associated with outcome.

Variable	Unadjusted model		Adjusted model 1^†^		Adjusted model 2^‡^	
	OR, 95% (CI)	*p* value	OR, 95% (CI)	*p* value	OR, 95% (CI)	*p* value
Age	1.06 (1.02, 1.09)	<0.001	1.03 (0.99, 1.07)	0.117	1.04 (1.00, 1.08)	0.048
Diabetes	3.78 (1.51, 9.48)	0.005	3.15 (1.09, 9.09)	0.034	2.49 (0.89, 6.94)	0.082
Current drinker	0.31 (0.13, 0.73)	0.008	0.21 (0.07, 0.66)	0.008	0.30 (0.11, 0.86)	0.025
Systolic blood pressure	1.01 (1.00, 1.03)	0.037	1.01 (0.99, 1.02)	0.478	1.01 (0.99, 1.02)	0.315
Baseline NIHSS	1.07 (1.01, 1.12)	0.014	1.05 (0.99, 1.11)	0.127	1.06 (1.00, 1.13)	0.038
Successful recanalization	0.16 (0.04, 0.76)	0.021	0.08 (0.01, 0.52)	0.009	0.10 (0.02, 0.69)	0.019
log₂(IL-6) (per doubling)	1.45 (1.20, 1.75)	<0.001	1.43 (1.14, 1.79)	0.002	–	–
log₂(IL-10) (per doubling)	2.43 (1.45, 4.06)	<0.001	–	–	1.86 (1.02, 3.36)	0.041

CI, confidence interval; NIHSS, National Institutes of Health Stroke Scale; OR, odds ratio; IL, interleukin.

^†^Adjusted Model 1 included age, diabetes, current drinker, systolic blood pressure, baseline NIHSS, successful recanalization, and log₂-transformed IL-6.

^‡^Adjusted Model 2 included age, diabetes, current drinker, systolic blood pressure, baseline NIHSS, successful recanalization, and log₂-transformed IL-10.

To evaluate the independent contributions of IL-6 and IL-10, two adjusted models were constructed. In Adjusted Model 1, which incorporated clinical variables and log₂-transformed IL-6, diabetes (OR = 3.15, 95% CI: 1.09–9.09, *p* = 0.034), current alcohol consumption (OR = 0.21, 95% CI: 0.07–0.66, *p* = 0.008), baseline NIHSS score (OR = 1.05, 95% CI: 0.99–1.11, *p* = 0.127), and successful recanalization (OR = 0.08, 95% CI: 0.01–0.52, *p* = 0.009) remained associated with outcomes. Notably, log₂-transformed IL-6 was identified as an independent predictor (OR = 1.43 per doubling, 95% CI: 1.14–1.79, *p* = 0.002). In Adjusted Model 2, which replaced IL-6 with IL-10, log₂-transformed IL-10 was also significantly associated with poor outcome (OR = 1.86 per doubling, 95% CI: 1.02–3.36, *p* = 0.041). Furthermore, variance inflation factor (VIF) analysis was performed to evaluate potential multicollinearity. All VIF values were less than 1.3 in both Adjusted Model 1 and Model 2 (with VIF values of 1.21 and 1.16 for age, and 1.28 and 1.21 for log₂-IL-6 and log₂-IL-10, respectively), demonstrating the absence of significant multicollinearity. Thus, the slight fluctuation in the significance of age between the two models represents minor statistical variation rather than collinearity-induced instability.

### Model development and discriminative performance

To assess the incremental predictive value of inflammatory cytokines, three models were constructed: Clinical model (include age, systolic blood pressure, baseline NIHSS score, current drinker, diabetes, and successful recanalization), Cytokine model (include IL-6 and IL-10) and Combined model.

**Figure 2:**
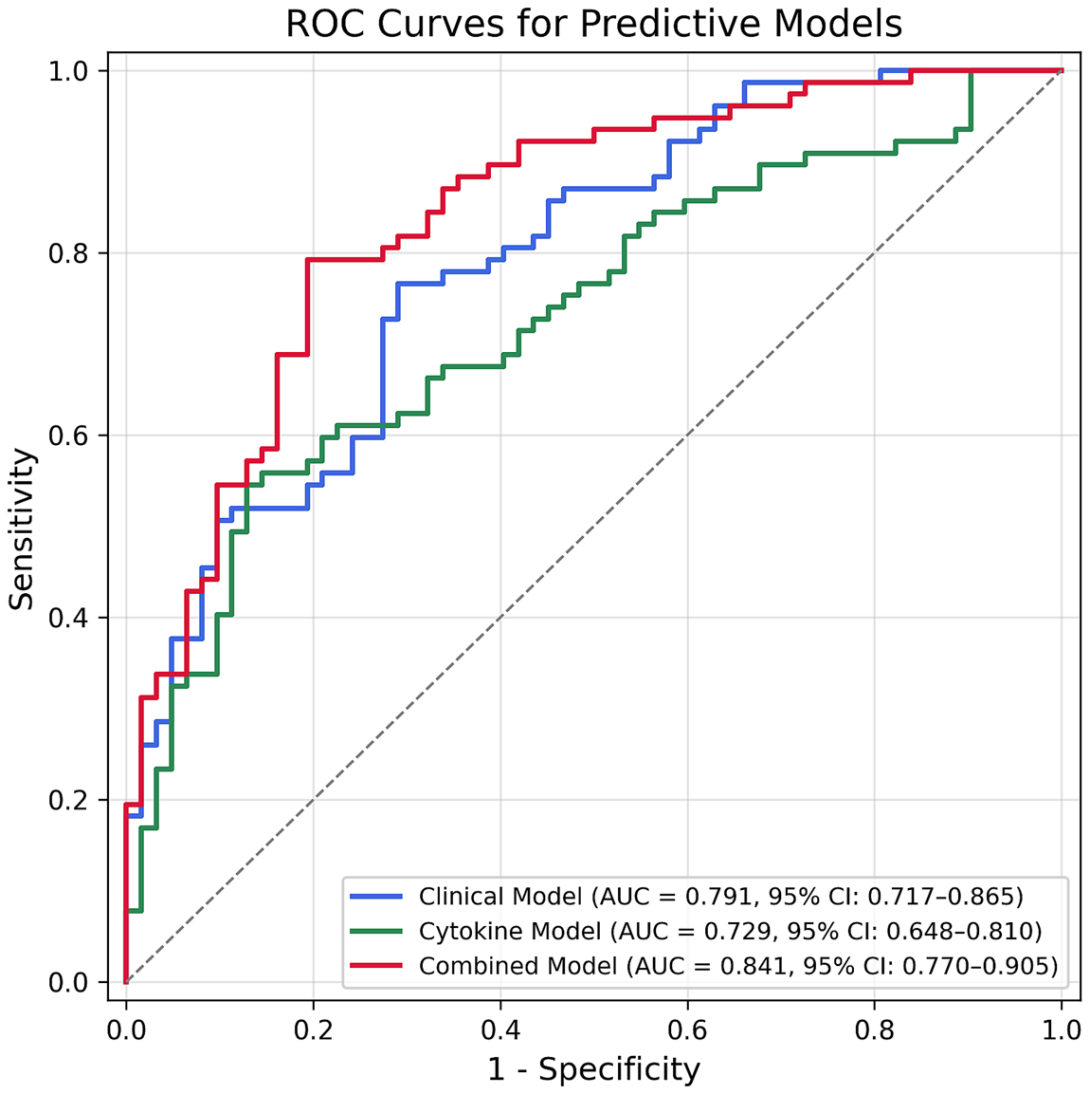
Receiver operating characteristic (ROC) curves comparing the predictive performance of different models for 90-day poor functional outcome. Abbreviations: AUC, area under the curve; CI, confidence interval; The blue line represents the clinical model, which incorporated age, systolic blood pressure, baseline NIHSS, current drinker, diabetes, and successful recanalization (AUC = 0.791). The green line represents the cytokine model, which included log₂-transformed IL-6 and IL-10 (AUC = 0.729). The red line represents the combined model, which integrated both the aforementioned clinical characteristics and cytokines (AUC = 0.841). The DeLong test demonstrated that the combined model achieved a statistically significant improvement in discriminative ability compared with the clinical model (*p* = 0.015).

Receiver operating characteristic (ROC) curve analysis demonstrated that the combined model achieved the highest discriminative performance, with an AUC of 0.841 (95% CI: 0.770–0.905), compared with 0.791 (95% CI: 0.717–0.865) for the clinical model and 0.729 (95% CI: 0.648–0.810) for the cytokine model (Figure 2).

The improvement in AUC between the combined model and the clinical model was statistically significant (DeLong test, *p* = 0.015). Although the absolute increase in AUC was moderate, it indicates a meaningful enhancement in predictive accuracy.

### Calibration and clinical utility

**Figure 3:**
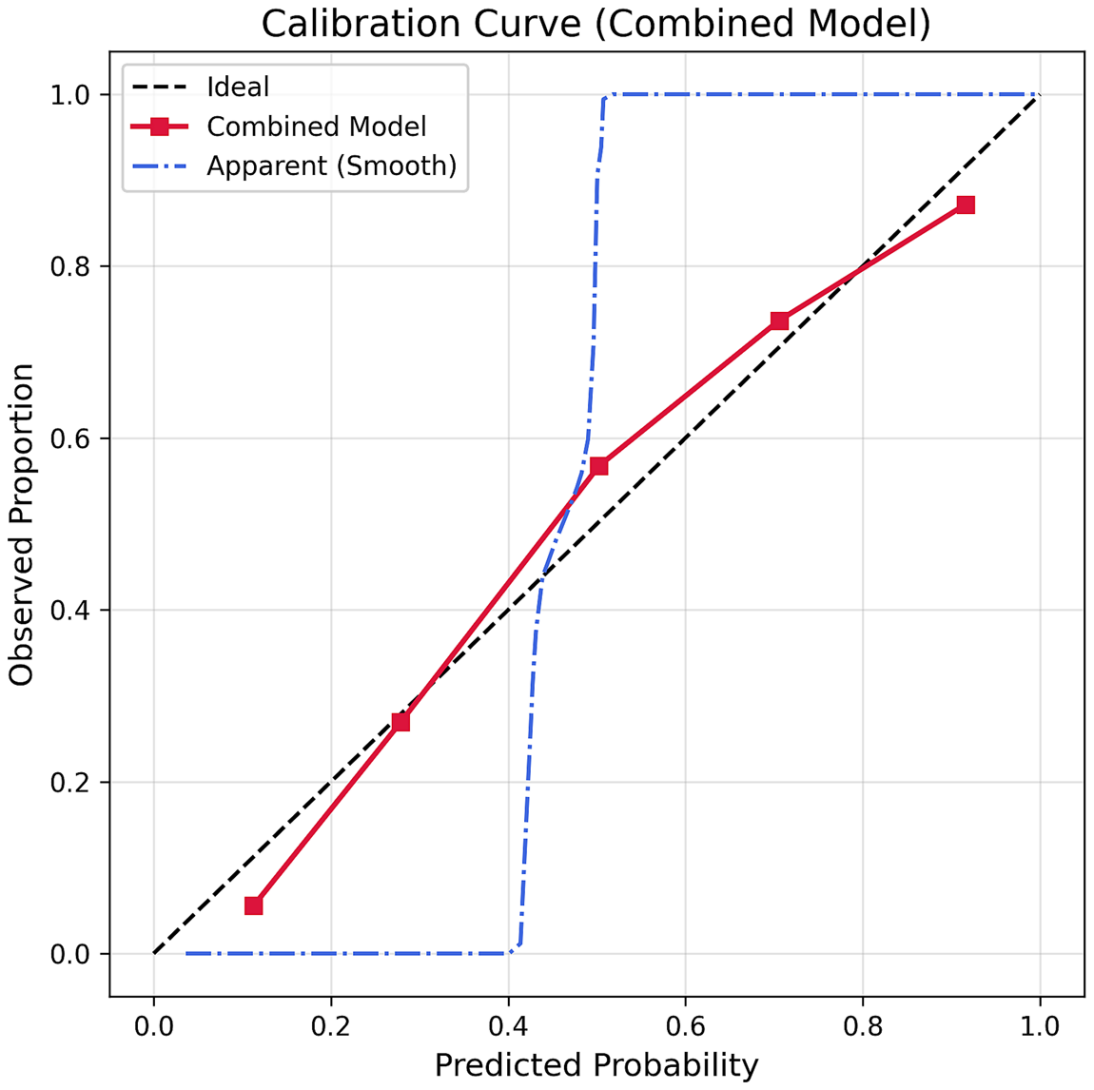
Calibration curve of the combined model for predicting 90-day poor functional outcome. The *x*-axis represents the predicted probability, and the *y*-axis represents the observed proportion of poor functional outcome. The diagonal black dashed line indicates perfect prediction (ideal model). The blue dash-dotted line represents the apparent calibration curve of the model, while the red solid line with square markers represents the calibration performance of the combined model.

Model calibration was assessed using the Hosmer–Lemeshow goodness-of-fit test and calibration curves. Both the clinical model (*p* = 0.770) and the combined model (*p* = 0.298) demonstrated good agreement between predicted and observed outcomes, suggesting adequate model fit within the internal validation framework. The calibration curve of the combined model further confirmed excellent concordance (Figure 3).

**Figure 4:**
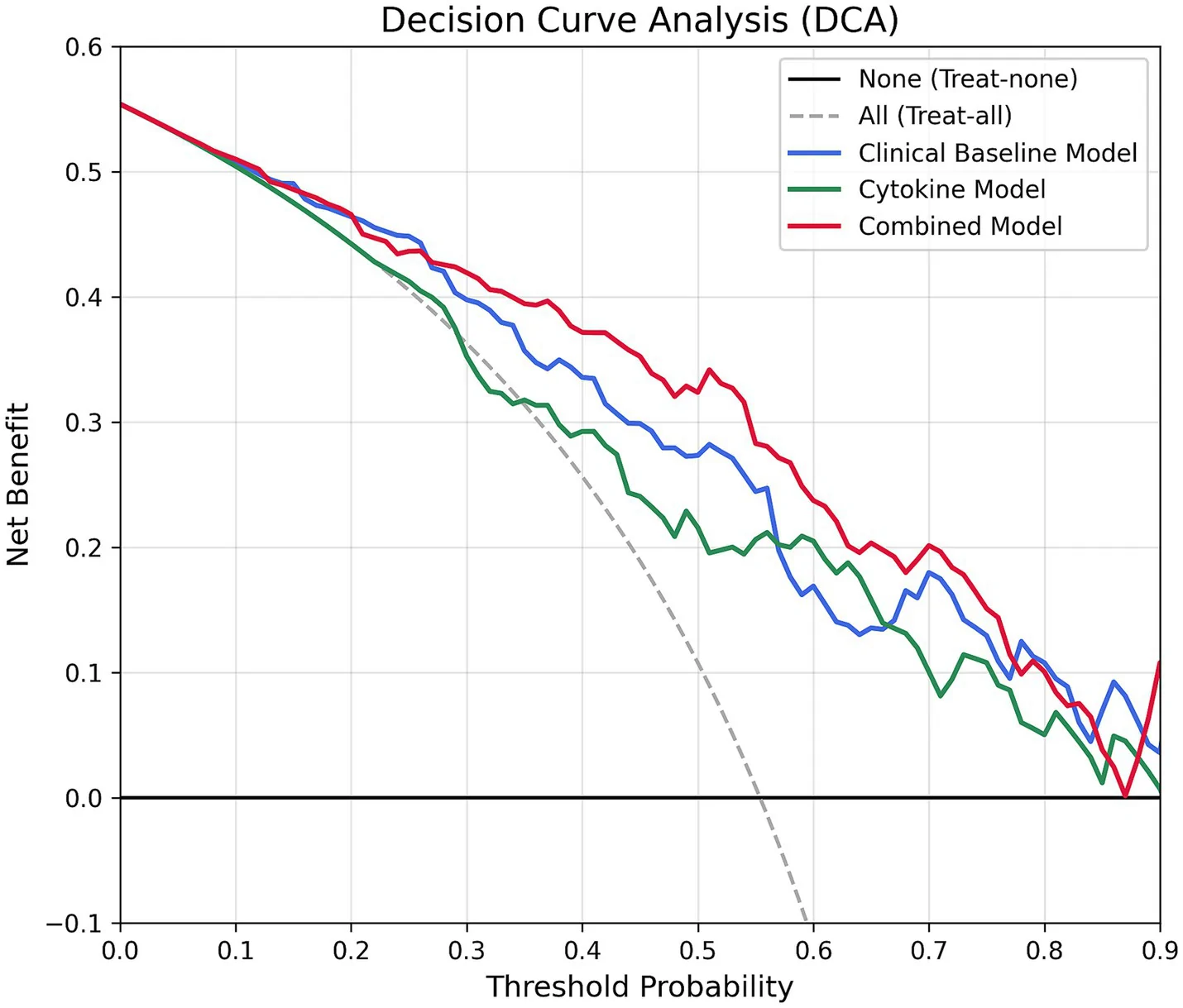
Decision curve analysis (DCA) evaluating the clinical utility of the predictive models. The *x*-axis indicates the threshold probability, and the *y*-axis indicates the net clinical benefit. The horizontal black line assumes that no patients will experience a poor functional outcome (treat-none strategy), while the grey line assumes that all patients will experience a poor functional outcome (treat-all strategy). The red, blue, and green curves represent the combined model, clinical model, and cytokine model, respectively. The combined model provided the highest net clinical benefit across a wide range of threshold probabilities.

Decision curve analysis (DCA) showed that the combined model provided a higher net clinical benefit across a wide range of threshold probabilities compared with both the clinical model and default strategies (treat-all or treat-none) (Figure 4), indicating its potential value in guiding individualized clinical decision-making.

### Incremental predictive value: reclassification and discrimination

To further quantify the added predictive value of inflammatory cytokines, net reclassification improvement (NRI) and integrated discrimination improvement (IDI) were calculated (Table 3).

**Table 3:** Reclassification and discrimination statistics for 90-day poor functional outcome after endovascular treatment by inflammatory cytokines.

Model	AUC-ROC	H–M		NRI			IDI		
	Estimate (95% CI)	*χ* ^2^	*p* value	Estimate (95% CI)	*Z*	*p* value	Estimate (95% CI)	*Z*	*p* value
Clinical model	0.791 (0.717–0.865)	4.887	0.770	Reference	–	–	Reference	–	–
Combined model	0.841 (0.770–0.905)	9.547	0.298	43.32 (10.82–75.81)	2.613	0.009	7.27 (3.06–11.49)	3.390	0.0007

AUC, area under the curve; ROC, receiver operating characteristic curve; CI, confidence interval; H–M, Hosmer–Lemeshow *χ*^2^ test; IDI, integrated discrimination improvement; NIHSS, National Institutes of Health Stroke Scale; NRI, net reclassification index.

Clinical model included age, systolic blood pressure, baseline NIHSS, current drinker, diabetes, and successful recanalization.

Combined model included Clinical model plus log₂-transformed IL-6 and IL-10.

The addition of log₂-transformed IL-6 and IL-10 to the clinical model resulted in a significant improvement in risk reclassification, with a continuous NRI of 43.32% (95% CI: 10.82–75.81%, *p* = 0.009). Similarly, the IDI increased by 7.27% (95% CI: 3.06–11.49%, *p* = 0.0007), indicating enhanced discrimination ability. The absolute improvement in IDI was 7.27%.

These findings suggest that incorporating inflammatory cytokines substantially improves individual risk stratification. However, given the relatively limited sample size, these improvements should be interpreted with appropriate caution.

### Sensitivity analysis

Furthermore, in the sensitivity analysis for internal validation, the optimism-corrected AUC of the combined model remained robust at 0.802 after 1,000 bootstrap resamples. The minor optimism estimate (0.039) between the apparent and corrected AUC indicates that the combined model was not severely overfitted and maintained stable predictive ability.

## Discussion

In this prospective cohort of patients with acute ischemic stroke undergoing endovascular treatment (EVT), we systematically evaluated the association between inflammatory cytokines and 90-day functional outcomes and developed a predictive model integrating clinical variables and inflammatory markers. The results showed that serum IL-6 was an independent predictor of poor functional outcome at 90 days, whereas IL-10 did not retain independent prognostic significance in multivariable analysis. In addition, the combined model integrating inflammatory cytokines demonstrated superior discrimination, calibration, and clinical net benefit compared with the clinical model alone, suggesting that inflammatory markers may further improve prognostic assessment beyond conventional clinical variables.

Inflammation plays a central role in the pathophysiology of acute ischemic stroke. Following cerebral ischemia, microglia are rapidly activated and release a variety of pro- and anti-inflammatory cytokines, forming a complex regulatory network (3, 4, 12). IL-6, a prototypical pro-inflammatory cytokine, contributes to blood–brain barrier disruption, leukocyte recruitment, and neuronal injury (13, 14), whereas IL-10 primarily exerts anti-inflammatory effects by suppressing pro-inflammatory signaling and modulating immune responses (15, 16). Thus, cytokine levels reflect both the intensity of inflammatory responses and the host’s regulatory mechanisms following brain injury.

Our finding that IL-6 is independently associated with poor functional outcome is consistent with previous studies. Whiteley et al. identified IL-6 as one of the most robust inflammatory markers associated with mortality and poor functional outcome after stroke in a systematic review (17). Bustamante et al. further demonstrated in a prospective cohort that IL-6 independently predicts 90-day functional outcomes (7).

Mechanistically, IL-6 amplifies inflammatory cascades and exacerbates brain injury. Chamorro et al. highlighted that IL-6 promotes inflammation and blood–brain barrier disruption via the JAK/STAT signaling pathway (3). In the EVT setting, reperfusion may restore blood flow but also induce reperfusion injury and inflammatory activation (18). Therefore, the independent predictive role of IL-6 observed in this study likely reflects the combined effects of ischemic injury and reperfusion-associated inflammation.

In Adjusted Model 2, log₂-transformed IL-10 was significantly associated with 90-day poor functional outcome (OR = 1.86 per doubling, 95% CI: 1.02–3.36, *p* = 0.041). However, in the combined model where both cytokines were entered simultaneously, log₂-transformed IL-10 did not retain independent prognostic significance (OR = 1.21, 95% CI: 0.63–2.32, *p* = 0.564). This attenuation of IL-10’s effect in the presence of IL-6 may reflect the stronger prognostic dominance of IL-6 in this limited sample, although VIF analysis did not indicate significant multicollinearity. Vila et al. reported that elevated IL-10 may reflect either protective anti-inflammatory responses or compensatory immunoregulation in severe inflammation (19). Moreover, systematic reviews have shown inconsistent associations between IL-10 and stroke outcomes. We must also acknowledge that the relatively limited sample size (*N* = 139) in this study might lead to statistical instability and limit the power to fully disentangle the independent effects of these correlated biomarkers. Larger multicenter cohorts are warranted to clarify the independent prognostic role of IL-10 relative to IL-6.

Furthermore, our study demonstrates that incorporating inflammatory biomarkers into conventional clinical models modestly improves prognostic performance. Specifically, the combined model showed a statistically significant improvement in discrimination compared with the clinical model alone (AUC: 0.841 vs. 0.791; DeLong test *p* = 0.015). In addition, the inclusion of cytokines resulted in a marked improvement in risk reclassification, with a continuous net reclassification improvement (NRI) of 43.32% (*p* = 0.009) and an integrated discrimination improvement (IDI) of 7.27% (*p* = 0.0007). Although the absolute increase in IDI was 7.27%, its robust statistical significance, coupled with the substantial improvement in net reclassification, suggests that inflammatory biomarkers provide meaningful incremental prognostic value beyond conventional clinical predictors. However, the clinical relevance of such a gain should be interpreted with caution. To date, although various prognostic models based on peripheral blood markers have been proposed to predict outcomes after EVT, few have comprehensively integrated key upstream inflammatory cytokines into conventional clinical risk scoring systems (20, 21). Traditional predictors, including age, baseline NIHSS score, and successful recanalization, primarily reflect the severity of initial neurological injury and procedural efficacy. In contrast, inflammatory cytokines such as IL-6 and IL-10 capture secondary injury processes and the host immune response following reperfusion, thereby providing complementary prognostic information. Nonetheless, the large magnitude of continuous NRI (43.32%) relative to the AUC improvement (0.050) should be interpreted with caution, as continuous NRI is sensitive to minor probability shifts and prone to overestimation in relatively small samples (*N* = 139). Future large-scale multicenter studies are required to validate the precise clinical utility of these markers.

The clinical implications of these findings are substantial. Early identification of patients at high risk of poor functional outcome may facilitate tailored management strategies, including intensified monitoring and optimization of post-procedural care. Although anti-inflammatory therapies in stroke remain largely investigational, accumulating evidence suggests that targeting specific inflammatory pathways may improve outcomes (5, 22). Given the observational design of this study, no direct therapeutic recommendations can be made. However, inflammation-based risk stratification may help identify patients for future trials of immunomodulation.

Several limitations of this study should be acknowledged. First, the sample size was relatively limited, and the inclusion rate was low (13.9%, 139/1003). It is important to note that cytokine testing was not a mandated protocol in the prospective registry, and the data were retrieved retrospectively. The missing data were primarily driven by objective logistical factors, such as the intermittent unavailability of laboratory consumables and hospital testing policy changes, rather than patient clinical severity. Consequently, the missingness of cytokine data is likely random, which mitigates the risk of directional selection bias. To empirically evaluate this missing-data assumption, we compared the baseline characteristics of included (*n* = 139) and excluded (*n* = 848) patients with available registry records (representing 98.1% [848/864] of the total excluded cohort, with 16 patients omitted due to incomplete baseline records in the registry). As shown in Supplementary Table S1 (available as Supplementary material), no significant differences were observed across major clinical and demographic variables, including age, sex, baseline stroke severity, and successful recanalization rates, supporting that the missingness of cytokine data did not introduce major directional selection bias. Second, the study was derived from a single participating center of the DETECT2-China registry, which limits external validity. Third, although internal bootstrap validation demonstrated a robust optimism-corrected AUC of 0.802, the model was not externally validated in an independent cohort. Therefore, the reported performance metrics might still be optimistic, and the generalizability of the combined model needs to be rigorously confirmed in future multicenter, large-scale studies. Additionally, while we restricted the number of predictors in our main adjusted models (Adjusted Models 1 and 2) to a maximum of 7 to maintain an events-per-variable (EPV) ratio of approximately 8.9, the Combined model incorporated both cytokines alongside the 6 clinical variables (totaling 8 predictors), resulting in a slightly lower EPV ratio of approximately 7.75. Although this slightly falls below the standard threshold of 10, it was methodologically necessary to evaluate the joint prognostic value of both cytokines, and bootstrap validation demonstrated robust performance, indicating that overfitting was minimal. Fourth, cytokine levels were measured only at a single time point (post-EVT), which may not adequately capture the dynamic evolution of post-stroke inflammatory responses. Serial measurements could provide more comprehensive insights. Fifth, “current drinker” status was analyzed as a binary variable (“current drinker” vs. “non-current drinker”) without detailed information on alcohol type, frequency, or quantity. The observed “protective” association is biologically counterintuitive and is highly likely a spurious finding due to statistical coincidence (arising from our relatively small sample size, *N* = 139) or residual confounding by unmeasured health-related or lifestyle factors. Future larger cohorts with detailed drinking profiles are needed to clarify this association (23, 24). Sixth, detailed data on concurrent chronic inflammatory or autoimmune diseases were not systematically collected in the registry. Although patients with severe active infections were clinically excluded from EVT, the potential confounding effect of undocumented subclinical or chronic inflammatory conditions on cytokine levels cannot be entirely ruled out. Finally, despite multivariable adjustment, residual confounding cannot be entirely excluded, particularly from unmeasured factors such as infarct volume, collateral status, or postprocedural complications.

## Conclusion

In conclusion, IL-6 is independently associated with poor 90-day functional outcome in AIS patients undergoing EVT. Adding these inflammatory cytokines to a conventional clinical model modestly improves risk discrimination and reclassification. However, given the lack of external validation, these findings warrant cautious interpretation, and future large-scale multicenter studies are needed to confirm their clinical utility.

## Data Availability

The original contributions presented in the study are included in the article/Supplementary material, further inquiries can be directed to the corresponding author/s.

## References

[1] Goyal M Menon BK van Zwam WH. Endovascular thrombectomy after large-vessel ischaemic stroke: a meta-analysis of individual patient data from five randomised trials. Lancet. (2016) 387:1723–31. doi: 10.1016/S0140-6736(16)00163-X, 26898852

[2] Hilkens NA Casolla B Leung TW de Leeuw FE. Stroke (seminar). Lancet. (2024) 403:2820–36. doi: 10.1016/s0140-6736(24)00642-1 38759664

[3] Iadecola C Anrather J. The immunology of stroke: from mechanisms to translation. Nat Med. (2011) 17:796–808. doi: 10.1038/nm.2399, 21738161 PMC3137275

[4] Anrather J Iadecola C. Inflammation and stroke: an overview. Neurotherapeutics. (2016) 13:661–70. doi: 10.1007/s13311-016-0483-x, 27730544 PMC5081118

[5] Chamorro Á Dirnagl U Urra X Planas AM. Neuroprotection in acute stroke: targeting excitotoxicity, oxidative and nitrosative stress, and inflammation. Lancet Neurol. (2016) 15:869–81. doi: 10.1016/s1474-4422(16)00114-9, 27180033

[6] Smith CJ Emsley HC Gavin CM Georgiou RF Vail A Barberan EM . Peak plasma interleukin-6 and other peripheral markers of inflammation in the first week of ischaemic stroke correlate with brain infarct volume, stroke severity and long-term outcome. BMC Neurol. (2004) 4:2. doi: 10.1186/1471-2377-4-2, 14725719 PMC331413

[7] Bustamante A Sobrino T Giralt D García-Berrocoso T Llombart V Ugarriza I . Prognostic value of blood interleukin-6 in the prediction of functional outcome after stroke: a systematic review and meta-analysis. J Neuroimmunol. (2014) 274:215:224. doi: 10.1016/j.jneuroim.2014.07.015, 25091431

[8] Asowata OJ Okekunle AP Olaiya MT Akinyemi J Owolabi M Akpa OM. Stroke risk prediction models: a systematic review and meta-analysis. J Neurol Sci. (2024) 460:122997. doi: 10.1016/j.jns.2024.122997 38669758

[9] Brott T Adams HP Jr Olinger CP Marler JR Barsan WG Biller J . Measurements of acute cerebral infarction: a clinical examination scale. Stroke. (1989) 20:864–70. doi: 10.1161/01.str.20.7.864, 2749846

[10] van Swieten JC Koudstaal PJ Visser MC Schouten HJ van Gijn J. Interobserver agreement for the assessment of handicap in stroke patients. Stroke. (1988) 19:604–7. doi: 10.1161/01.str.19.5.604, 3363593

[11] Zaidat OO Yoo AJ Khatri P Tomsick TA von Kummer R Saver JL . Recommendations on angiographic revascularization grading standards for acute ischemic stroke: a consensus statement. Stroke. (2013) 44:2650–63. doi: 10.1161/strokeaha.113.001972 23920012 PMC4160883

[12] Kumari S Dhapola R Sharma P Nagar P Medhi B HariKrishnaReddy D. The impact of cytokines in neuroinflammation-mediated stroke. Cytokine Growth Factor Rev. (2024) 78:105–19. doi: 10.1016/j.cytogfr.2024.06.002, 39004599

[13] Armstead WM Hekierski H Pastor P Yarovoi S Higazi AA Cines DB. Release of IL-6 after stroke contributes to impaired cerebral autoregulation and hippocampal neuronal necrosis through NMDA receptor activation and upregulation of ET-1 and JNK. Transl Stroke Res. (2019) 10:104–11. doi: 10.1007/s12975-018-0617-z, 29476447

[14] Pawluk H Woźniak A Tafelska-Kaczmarek A Kosinska A Pawluk M Sergot K . The role of IL-6 in ischemic stroke. Biomolecules. (2025) 15:470. doi: 10.3390/biom15040470, 40305179 PMC12024898

[15] Wu W Luo Z Shen D Lan T Xiao Z Liu M . IL-10 protects against OPC ferroptosis by regulating lipid reactive oxygen species levels post stroke. Redox Biol. (2024) 69:102982. doi: 10.1016/j.redox.2023.102982, 38070317 PMC10755589

[16] Izzy S Yahya T Albastaki O Abou-el-Hassan H Aronchik M Cao T . Nasal anti-CD3 monoclonal antibody ameliorates traumatic brain injury, enhances microglial phagocytosis and reduces neuroinflammation via IL-10-dependent T(reg)-microglia crosstalk. Nat Neurosci. (2025) 28:499–516. doi: 10.1038/s41593-025-01877-7, 40016353 PMC11893472

[17] Whiteley W Jackson C Lewis S Lowe G Rumley C Sandercock P . Inflammatory markers and poor functional outcome after stroke: a prospective cohort study and systematic review of interleukin-6. PLoS Med. (2009) 6:e1000145. doi: 10.1371/journal.pmed.1000145 19901973 PMC2730573

[18] Bendszus M Fiehler J Subtil F Bonekamp S Aamodt AH Fuentes B . Endovascular thrombectomy for acute ischaemic stroke with established large infarct: multicentre, open-label, randomised trial. Lancet. (2023) 402:1753–63. doi: 10.1016/s0140-6736(23)02032-9, 37837989

[19] Vila N Castillo J Dávalos A Chamorro A. Proinflammatory cytokines and early neurological worsening in ischemic stroke. Stroke. (2000) 31:2325–9. doi: 10.1161/01.str.31.10.2325, 11022058

[20] Deng QW Huang S Li S Zhai Q Zhang Q Wang ZJ . Inflammatory factors as potential markers of early neurological deterioration in acute ischemic stroke patients receiving endovascular therapy—the AISRNA study. J Inflamm Res. (2021) 14:4399–407. doi: 10.2147/jir.S317147, 34511974 PMC8421252

[21] Du M Xu L Zhang X Huang X Cao H Qiu F . Association between inflammatory burden index and unfavorable prognosis after endovascular thrombectomy in acute ischemic stroke. J Inflamm Res. (2023) 16:3009–17. doi: 10.2147/jir.S419087, 37489151 PMC10363388

[22] Kalogeris T Baines CP Krenz M Korthuis RJ. Cell biology of ischemia/reperfusion injury. Int Rev Cell Mol Biol. (2012) 298:229–317. doi: 10.1016/b978-0-12-394309-5.00006-7, 22878108 PMC3904795

[23] Reynolds K Lewis B Nolen JD Kinney GL Sathya B He J. Alcohol consumption and risk of stroke: a meta-analysis. JAMA. (2003) 289:579–88. doi: 10.1001/jama.289.5.579, 12578491

[24] Fillmore KM Stockwell T Chikritzhs T Bostrom A Kerr W. Moderate alcohol use and reduced mortality risk: systematic error in prospective studies and new hypotheses. Ann Epidemiol. (2007) 17:S16–23. doi: 10.1016/j.annepidem.2007.01.005, 17478320

