# Supplementary material for: Serum inflammatory cytokines predict 90-day outcome after endovascular treatment in acute ischemic stroke
Source: Front Neurol. 2026 Aug 26;17:1903651. doi: 10.3389/fneur.2026.1903651 (PMC13553452; doi:10.3389/fneur.2026.1903651)
Supplement: Supplementary file 1 [file Table_1.DOCX]

**Supplementary Material**

*Serum Inflammatory Cytokines Predict 90-Day Outcome After Endovascular Treatment in Acute Ischemic Stroke*

**Supplementary Table S1. Comparison of baseline characteristics between included and excluded patients**

| **Variable** | **Included Group (n=139)** | **Excluded Group (n=848)** | **P Value** |
| --- | --- | --- | --- |
| Age (years), mean ± SD | 64.68 ± 11.73 | 63.77 ± 11.55 | 0.394 |
| Male gender, n (%) | 101 (72.66%) | 631 (74.67%) | 0.690 |
| Smoking status, n (%) | 71 (51.08%) | 489 (57.87%) | 0.160 |
| Drinking status, n (%) | 30 (21.58%) | 476 (56.33%) | <0.001 |
| Hypertension, n (%) | 88 (63.31%) | 493 (58.41%) | 0.320 |
| Diabetes mellitus, n (%) | 32 (23.02%) | 194 (22.99%) | 1.000 |
| Atrial fibrillation, n (%) | 32 (23.02%) | 143 (16.94%) | 0.106 |
| Systolic blood pressure (mmHg), mean ± SD | 144.50 ± 29.61 | 136.12 ± 24.29 | 0.002 |
| Diastolic blood pressure (mmHg), mean ± SD | 83.37 ± 15.04 | 80.76 ± 15.08 | 0.059 |
| Baseline NIHSS score, median [IQR] | 15.0 [12.0-19.0] | 15.0 [12.0-19.0] | 0.712 |
| Successful recanalization, n (%) | 124 (89.21%) | 778 (92.51%)‡ | 0.245 |

*Note: Denominators vary slightly for some variables in the excluded group due to missing data in the registry. The denominator for the excluded comparison group is 848 (representing 98.1% [848/864] of the total 864 excluded cohort, with 16 patients omitted due to incomplete baseline registry records). ‡ Denominator for successful recanalization in the excluded group is 841 due to missing data on recanalization status.
SBP, systolic blood pressure; DBP, diastolic blood pressure; NIHSS, National Institutes of Health Stroke Scale; IQR, interquartile range; SD, standard deviation.*
